# Preparation and properties of Pb/Sn/Al laminated composite anode for zinc electrowinning

**DOI:** 10.1039/c8ra04977g

**Published:** 2018-08-15

**Authors:** Zhaohui Han, Peixian Zhu, Jianhua Liu, Sivasankar Koppala, Lei Xu, Libo Zhang, Chandrasekar Srinivasa Kannan

**Affiliations:** Faculty of Metallurgical and Energy Engineering, Kunming University of Science and Technology Kunming 650093 PR China kmustleixu@126.com; State Key Laboratory of Complex Nonferrous Metal Resources Clean Utilization, Kunming University of Science and Technology Kunming 650093 PR China liujianhua501050@163.com; Faculty of Material Science and Engineering, Kunming University of Science and Technology Kunming 650093 PR China; Chemical Engineering Department, The Petroleum Institute, Khalifa University of Science and Technology Abu Dhabi United Arab Emirates

## Abstract

The use of Pb/Sn/Al composite anode materials has been limited due to the thermodynamic immiscibility between Pb and Al sheets during the welding process. Thus, herein, Sn has been added between Pb and Al sheets to fabricate a Pb/Sn/Al laminated composite *via* vacuum hot-pressing welding (at a temperature of 230 °C for 12 h under 0.5 MPa). Furthermore, the interfacial microstructure and mechanical and electrical properties are investigated. Good metallurgical bonding has been realized due to the addition of Sn, and block α-Pb and a small amount of β-Sn solid solutions are also formed at the interface. In comparison with the Pb–Ag alloy anode, the Pb/Sn/Al laminated composite presents superior mechanical strength (73.9 MPa), and good electrical conductivity of the Pb/Sn/Al composite has been obtained due to its sandwich laminated structure. Moreover, the Pb/Sn/Al composite reduces the electrode reaction energy and improves the electrocatalytic activity of the electrode to reduce the bath voltage.

## Abstract 2

The use of Pb/Sn/Al composite anode materials has been limited due to the thermodynamic immiscibility between Pb and Al sheets during the welding process.

## Graphical abstract

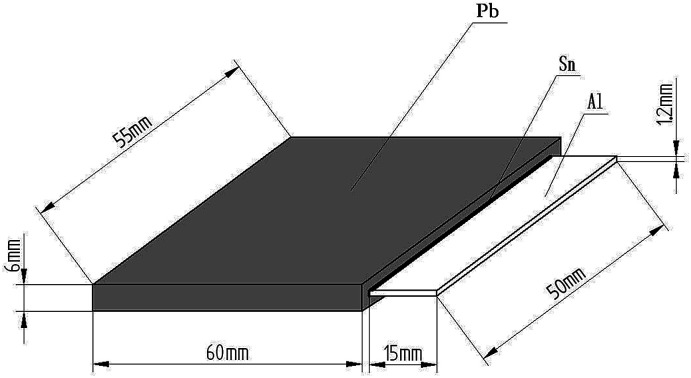

## 1. Introduction

Pb-based alloys, as preferred anode materials, are widely used in the wet metallurgy and electrochemistry industries because of their excellent properties including electrochemical properties and easy processing and recycling.^1^ Particularly, Pb-based alloys have never been replaced in the electrowinning processes for Zn, Cu, Ni, and Mn.^2–5^ Thus, improving the corrosion resistance, electrical conductivity, catalytic activity and mechanical strength of Pb-based alloy anode materials is a hot topic.^6,7^ Currently, Pb–(0.7–1.0 wt%)Ag binary alloys are used as the main anode materials in zinc electrowinning. However, they have several drawbacks including high silver content, low mechanical strength, high internal resistance, and relatively low electrochemical activity,^8,9^ which result in buckling and sagging of the anodes, uneven current distribution, and electrical short circuits within the electrolytic cell.^10^

To resolve the problems mentioned above, a great deal of research has been done. For example, alloy elements (Ca, Sn, and Co^11,12^) and rare earth elements (Ce, Tb, Yb, and Sm^13,14^) have been added to reduce the Ag content. The electrocatalytic activity increases after adding the elements, but the stabilization decreases. Also, electrocatalysts (IrO_2_ and RuO_2_ (ref. 15–17)) were deposited on the surface of Pb–(0.7–1.0 wt%)Ag alloys to enhance their surface electrocatalytic activity, which increased the production cost and decreased the service life. Under such circumstances, Pb/Al laminated composite electrode materials have been proposed in recent years.^18,19^ However, Pb and Al are immiscible, and they cannot be strongly combined *via* metallurgy bonding. To date, many researchers have focused on these immiscible alloys, but there has been no clear improvement in their properties.^20–22^ On the basis of thermodynamic calculations and phase diagrams, Sn has been considered as a good intermediary material to resolve the immiscibility between Pb and Al. Q. Jiang^23^ and L. Zhang^24^ prepared Pb/Sn/Al layered films and reported their interface microstructures, which illustrated that the addition of Sn played an important role in combining Pb and Al.

Herein, Pb/Sn/Al laminated composite anode materials have been prepared *via* vacuum hot-pressing welding at a temperature of 230 °C for 12 h under 0.5 MPa. The microstructure of the interface has been characterized in detail, and it has also been deeply analyzed *via* thermodynamic calculations. The results of electrical conductivity, electrocatalytic activity and bending strength of the composite are compared with those of the Pb–0.2 wt% Ag alloy anode.

## 2. Experimental

### 2.1 Material preparation

Commercial lead plates (1^#^ Pb, purity higher than 99.99%, thickness: 2.5 mm) were purchased from Yunnan Chihong Zn & Ge Co. Ltd., China, commercial aluminum plates (L2, purity higher than 99.9%, thickness: 1.2 mm) were purchased from Yunnan Aluminum Co. Ltd., China, commercial tin ingot (1^#^ Sn, purity higher than 99.9%) was purchased Yunnan Tin Group (Holding) Company Limited, China, and Pb–0.2 wt% Ag alloy was purchased Yunnan Daze Electrode Technology Co. Ltd., China.

Al sheets were surface treated prior to the experiments. They were polished using SiC abrasive papers and cleaned with deionized water. Subsequently, NaOH solution (10%) was used to remove the oxidation scale on their surface, which can influence the diffusion welding. Finally, the sheets were cleaned with acetone and deionized water and dried for use.

**Fig. 1:**
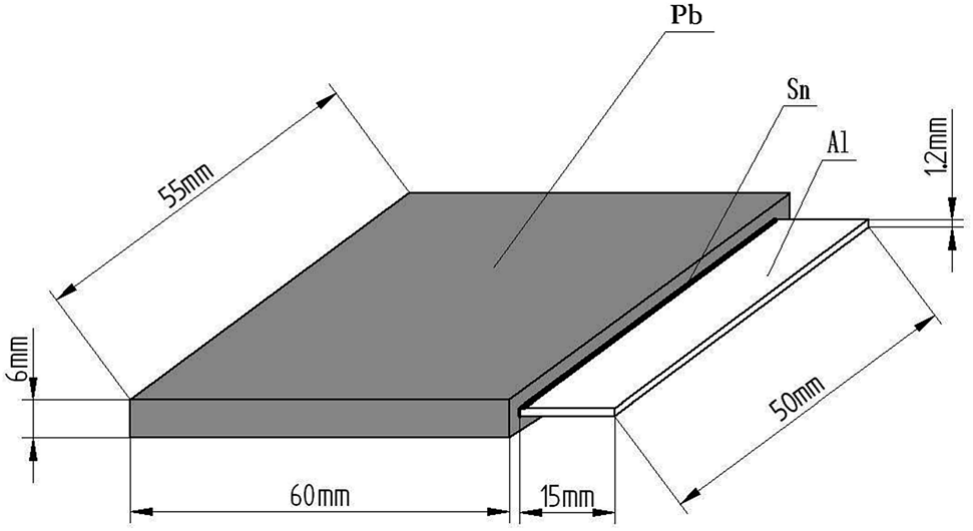
Schematic illustration of the Pb/Sn/Al laminated composite electrode material.

The Al sheets (after surface treatment) were placed in a plating bath (containing Sn solution) at 325 °C for 5–10 min to coat them with hot-dipped Sn (thickness of 30–50 μm). Then, Pb sheets and the Al sheets coated with hot-dipped Sn were placed in a vacuum hot-pressing diffusion welding stove at the heating temperature of 230 °C, pressure of 0.5 MPa, vacuum of 0.02 ppm, and hold time for 12 h to fabricate the Pb/Sn/Al composite material. A schematic illustration of the Pb/Sn/Al laminated composite anode material is presented in Fig. 1, which shows the sandwich structure in the Pb/Sn/Al laminated composite.

### 2.2 Material characterization

The cross-sectional area of the Pb/Sn/Al laminated composite was polished using conventional metallographic techniques to observe its morphology *via* scanning electron microscopy (SEM, XL-30). The chemical compositions of the transition layer were examined *via* energy dispersive spectroscopy (EDS). Also, the identification of phase was conducted *via* X-ray diffraction (XRD, D/MAX-3B) using Cu-K_α_ radiation (*λ* = 1.54060 Å) at a scanning speed of 10° min^−1^, scanning step width of 0.01°, working voltage of 200 kV and current of 200 mA. Three-point bending strength tests were carried out on a universal material testing machine (AG-IS). The electrical resistivity of the Pb/Sn/Al composite and Pb–Ag alloys (for comparison) was tested using the four-point probe method (as shown in Fig. 1), where silver wire was used as the probe and a multimeter (Keithley2182A) was used for the measurements. The *I*–*V* curves of the Pb/Sn/Al composite and Pb–Ag alloys were obtained using the linear scanning method with an electrochemistry workstation (CHI660-E), where the counter electrode was a platinum sheet, the reference electrode was a saturated calomel electrode (SCE), and the electrolyte was H_2_SO_4_ solution (0.5 mol L^−1^); the scan rate was 0.5 × 10^−2^ V s^−1^.

## 3. Results and discussion

### 3.1 Interfacial morphology and composition analysis

**Fig. 2:**
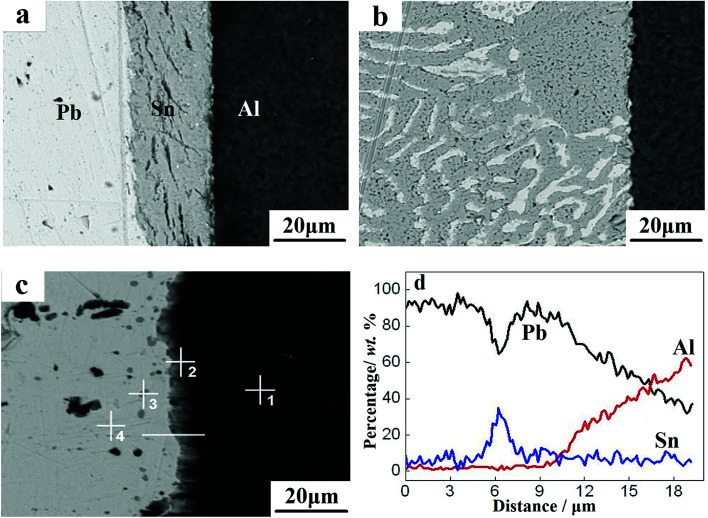
Cross-sectional morphology at different holding times: (a) 0 h, (b) 1 h, and (c) 12 h. (d) Line scanning results corresponding to (c).

The interfacial morphology of the Pb/Sn/Al composite is shown in Fig. 2. For the Pb/Sn/Al composite without heat treatment, as shown in Fig. 2a, a clear boundary remained between Al and Sn. With an increase in the holding time to 1 h (as presented in Fig. 2b), the diffusion dissolution reaction started between the Sn and Pb sheets to form a net texture. However, this net texture was not observed between the Sn and Al sheets. In addition, cracks appeared at the interface between the Sn and Al sheets. Upon increasing the holding time to 12 h, it was seen that a continuous transition layer (with a width of 3.4 μm) formed at the Al side (as shown in Fig. 2c). Furthermore, Fig. 2d presents the line scanning results of the transition layer in Fig. 2c, which indicated that Pb–Sn–Al metallurgical bonding was formed for a holding time of 12 h. Meanwhile, many gray particles and light blocks appeared at the Pb side. Thus, to identify the chemical composition of this substance, a point analysis was conducted (in Fig. 2c), and the results are presented in Table 1, which show that Sn did not diffuse into the Al side (point 1). However, at the transition layer (point 2), the contents of Pb, Sn and Al were 21.45%, 70.14% and 8.41%, respectively, which confirmed that the Pb–Sn–Al metallurgical bonding was well formed. For the gray particles (point 3), the contents of Pb and Sn were found to be 23.88% and 76.12%, respectively; correspondingly, the values were 89.92% and 10.08% for the light block (point 4). In this case, by combining the Al–Sn and Sn–Pb binary phase diagrams^25–27^ with the contents at points 3 and 4, the gray particles and light blocks could be preliminary identified as β-Sn and α-Pb solid solutions, respectively. Briefly, metallurgical bonding between the Pb and Al sheets was realized by adding intermediary Sn, which solved the problem of immiscibility of Pb and Al. Also, large block α-Pb and small-particle β-Sn solid solutions were formed at the interface.

**Table 1:** EDS point analysis of the transition layer

Location	Element (wt%)		
	Pb	Sn	Al
1	—	—	100
2	21.45	70.14	8.41
3	23.88	76.12	—
4	89.92	10.08	—

### 3.2 Thermodynamic analysis of stable phase

According to the above analysis, β-Sn and α-Pb solid solutions were formed at the interface, which were derived from the concentration difference based on thermodynamics. On the basis of Arrhenius equation,^28^

1 *D* = *D*_0_e^−*Q*/*RT*^

where *D* is the diffusion coefficient, *D*_0_ is the frequency factor, *Q* is the diffusion activation energy, *R* is the Boltzmann's constant (8.314 J mol^−1^ K^−1^), and *T* is the temperature.

The values of *D*_*0*_ and *Q* (as illustrated in Table 2) were entered in the Arrhenius equation to calculate the self-diffusion coefficient, and it was found that the self-diffusion coefficient of Sn (5.3 × 10^−15^ m^2^ s^−1^) was much higher than that of Pb (0.8 × 10^−15^ m^2^ s^−1^) at 230 °C. This suggested that Sn diffused before the Pb side, and the eutectic reaction may not start until the eutectic concentration has been reached. Meanwhile, liquidation of the Pb–Sn alloy occurred, and the Pb–Sn liquidation layer became wider with an increase in the holding time to 12 h. This process is mainly dependent on the diffusion coefficient of Sn into Pb (2.6 × 10^−16^ m^2^ s^−1^). Also, the process of homogenization occurred simultaneously. Subsequently, the eutectic reaction was terminated if the concentration of Sn atoms in the liquidation layer reached that of the eutectic liquid ingredient. Under this circumstance, the width of the liquidation layer was the largest, and the interdiffusion solution between Pb and Sn was observed.

**Table 2:** Diffusion coefficient, *D*, of Pb and Sn

Element	*D*_0_/cm^2^ s^−1^	*Q*/kJ mol^−1^	*D*/m^2^ s^−1^
Sn	10.7	108.4	5.3 × 10^−15^
Pb	0.995	107.4	0.8 × 10^−15^
Sn into Pb	0.29	99.4	2.6 × 10^−16^

Moreover, the enthalpy of mixing for the Pb–Sn solid solution was calculated using Miedema's theory^29^ to determine the stable phase formed, which is expressed as follows:2
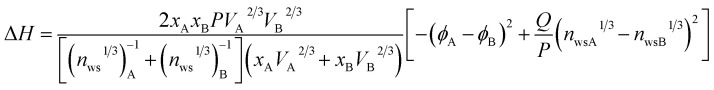
here, *x*_A_ and *x*_B_ are the mole fractions, *V*_A_ and *V*_B_ are the molar volumes, *n*_wsA_ and *n*_wsA_ are the electron concentrations on the boundary of the pure metal Wigner–Seitz, *ϕ*_A_ and *ϕ*_B_ are the chemical potentials, and *P* and *Q* are empirical constants (from Miedema's theory, Pb and Sn are non-transition elements, *P* = 10.6 and *Q*/*P* = 9.4). The enthalpy of mixing for the Pb–Sn solid solution can be calculated by entering the thermodynamic parameters (in Table 3) into eqn (2).

**Table 3:** Relevant thermodynamic parameters used for calculation^30^

Element	*n*_ws_^1/3^	*V* ^2/3^/cm^2^	*ϕ*
Sn	1.24	6.43	4.15
Pb	1.15	6.94	4.10

**Fig. 3:**
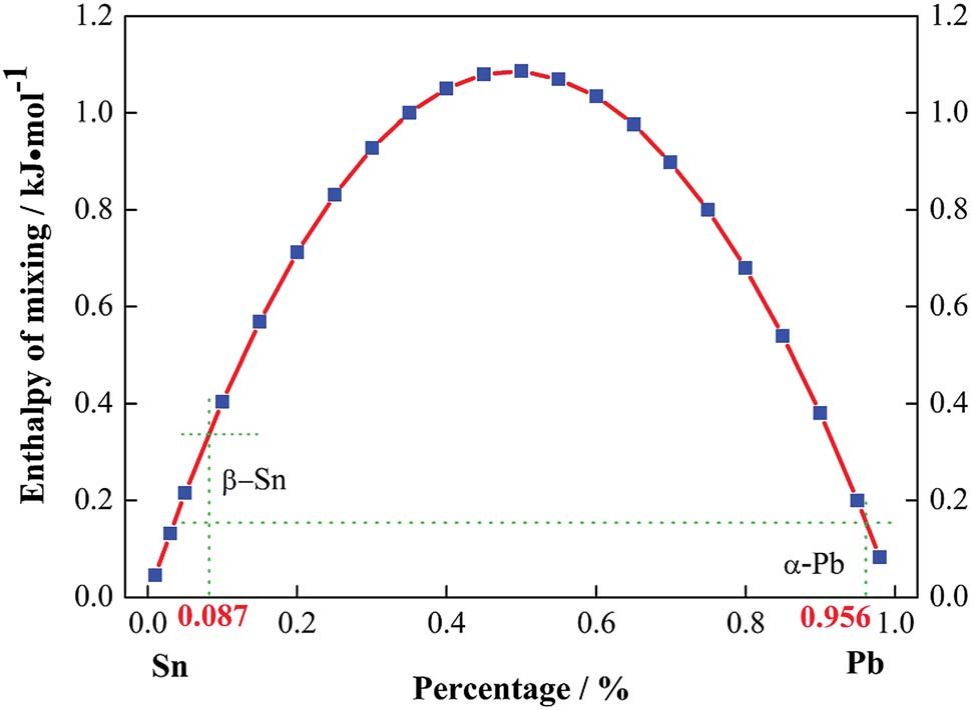
The enthalpy of mixing as a function of constituent percentage.

Fig. 3 illustrates the enthalpy of mixing for Pb–Sn alloys as a function of ingredient, where it can be seen that the α-Pb solid solution is more stable than the β-Sn solid solution. The difference between the enthalpies of mixing of the α-Pb and β-Sn solid solutions was about 0.3 kJ mol^−1^, which was in accordance with the result of ref. 31. Thus, a large amount of α-Pb solid solution remained with an increase in the holding time to 12 h, which was in good agreement with the above SEM observations.

**Fig. 4:**
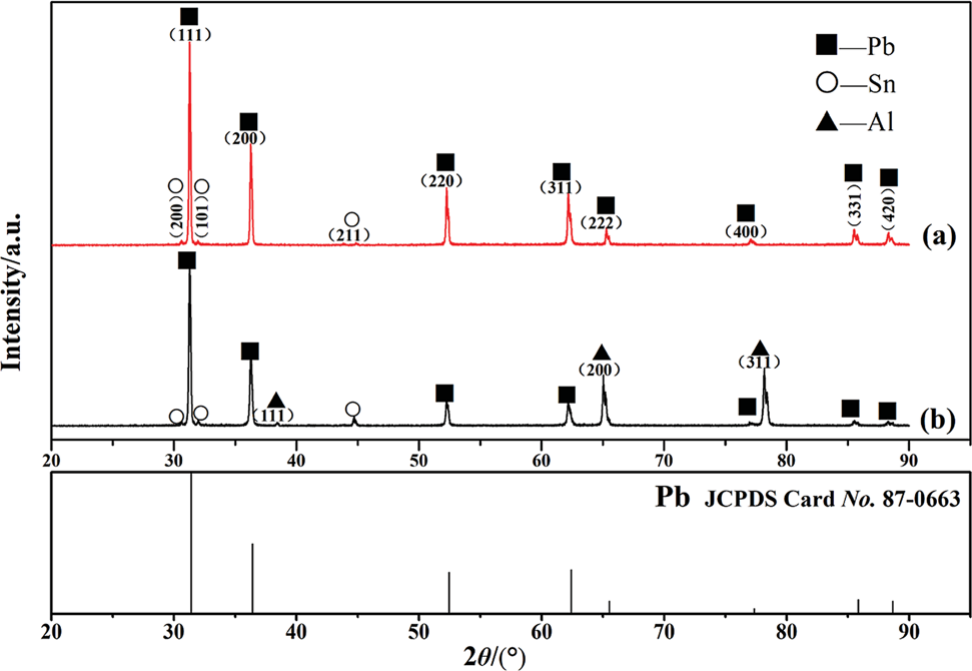
XRD patterns of the interface at the (a) Pb side and (b) Al side.

From the above analysis, metallurgical bonding was realized at the interface of the Pb/Sn/Al composite electrode material. Thus, XRD was used to identify the phase structure of the interface in the Pb/Sn/Al composite. According to the fabrication process of the Pb/Sn/Al composite, it is known that the thickness of Sn (30–50 μm) was very low for its accurate analysis. Therefore, the Pb/Sn/Al composite was separated *via* shear force into Al and Pb sides for characterization, and the results and standard atlas of Pb are shown in Fig. 4. Fig. 4a displays the XRD pattern of the Pb side, which shows that pure Pb and Sn coexisted, but no Al was present. Moreover, the diffraction peak of Sn was relatively weak and that of Pb slightly shifted to right compared with the XRD standard atlas. A possible reason for this was that the separation occurred at the Pb side, leaving residual stress on the surface, which led to a low content of Sn and a shift in the diffraction peak of Pb.

In the case of the Al side, the XRD pattern is shown in Fig. 4b. It can be observed that there were diffraction peaks for Pb, Al and a small amount of the Sn phase, indicating the Al–Sn solid solution was never produced at this condition. It is clear that the diffraction peaks of Pb shifted to right, and the reasons for this shift can be summarized as follows: on one hand, the Pb–Sn solid solution led to lattice distortion at the transition zone; on the other hand, residual stress remained on the surface of the Al side.

On the basis of Bragg equation (2*d* sin *θ* = *λ*), the diffraction peaks of Pb shifted to right, indicating that the interplanar spacing, *d*, decreased. This is because the Sn atoms (0.1862 nm) dissolved into the Pb (0.1935 nm) crystal, which led to lattice contraction and decrease in the interplanar spacing. It is inferred that the shifted diffraction peaks of Pb were closely related to the formation of the α-Pb solid solution.

In conclusion, a pure Al phase, α-Pb solid solution and Pb phase existed at the transition zone after a holding time of 12 h. However, the β-Sn phase (identified by point analysis) was not detected from the XRD patterns, which was because the amount of β-Sn was too low to detect.

### 3.3 Mechanical property analysis

**Fig. 5:**
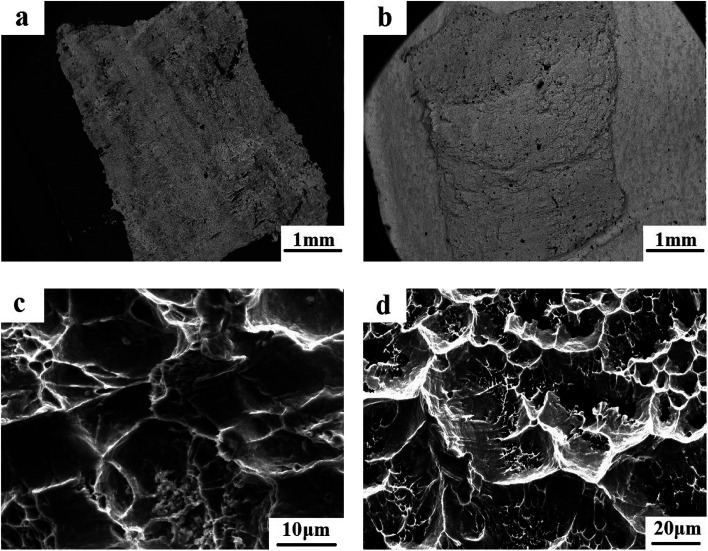
Fracture morphology of the transition zone: (a) and (c) for the Pb side, (b) and (d) for the Al side.

It is known that the fracture characteristics of the interfacial zone in the Pb/Sn/Al laminated composite play an important role in electrical performance and mechanical strength. Thus, herein, we observed the fracture feature at the interface *via* SEM using the backscattered model. The Pb/Sn/Al laminated composite was separated into the Pb side and Al side before observation. It was found that a pit, which formed during the procedure of tearing, remained on the Pb side (as shown Fig. 5a) and thus, it is reasonable to infer that fracture failure occurred at the Pb side but not at the Al side. Also, clear interlayers remained at the Al side (as shown in Fig. 5b), which were identified as Pb and a small amount of Pb–Sn solid solution from the above analysis, indicating good metallurgy bonding between the Pb and Sn sheets.

The magnified morphology of the fractured surface at the Pb side is shown in Fig. 5c, where round or elliptical dimples can be clearly observed, which provide strong evidence to confirm the ductile fracture at the interface. Furthermore, the cracks extended on the plane with different heights to form a tearing prism, presenting clear delaminated cracks. When the cleavage cracks extended at the grain boundary, the dimples and tear prisms were formed, which terminated the process of crack propagation. In addition, numerous microholes remained at the Al side, and there were clear “hunting slips” on the inner wall (as shown in Fig. 5d), indicating that the transition zone exhibited good ductility. Briefly, the fracture belonged to the typical ductile fracture, which was due to strong metallic bonding among atoms formed at the transition zone.

**Fig. 6:**
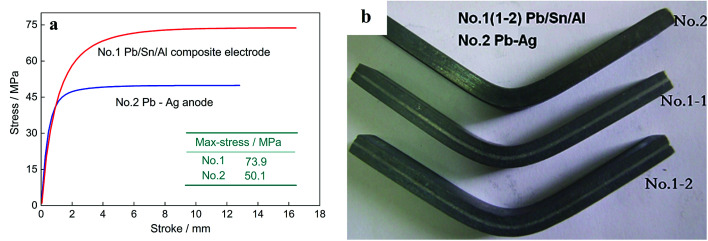
The curves of three-point bending tests for Pb/Sn/Al composite and Pb–Ag alloys (a) and the real image of the three-point bending of the test samples (b).

The bending strength of the Pb/Sn/Al laminated composite anode material is the key parameter to determine its service life and comprehensive properties. Fig. 6 shows the bending strength of the Pb/Sn/Al laminated composite electrodes and Pb–Ag alloy. No. 1-1 and 1-2 are the Pb/Sn/Al laminated composites treated under the same conditions, and the bending tests were performed several times to obtain the final results. At room temperature, the bending strength of the Pb/Sn/Al composite (73.9 MPa) was substantially higher than that of the Pb–Ag alloy (50.1 MPa), as shown in Fig. 6a. A real image of the bending test samples is presented in Fig. 6b. The good bending strength of the Pb/Sn/Al laminated composite electrode was due to its sandwich lamellar structure and continuous transition interface in comparison with that of the Pb–Ag alloy. On account of the addition of Sn, metallurgy bonding between the Pb and Al sheets was realized. During the bending tests, the synergistic effects contributed by the Al sheets, transition layer and Pb sheets efficiently transformed the load. Clearly, the sandwich structure and stabilization of the transition layer of the Pb/Sn/Al laminated composite electrode tremendously improved its bending strength, which can meet the requirements in the working environment.

### 3.4 Electrochemical analysis

The overpotential and catalytic activity of the electrode are directly influenced by the electrical conductivity of its matrix, where good electrical conductivity indicates low overpotential and fast chemical reaction; thus, the catalytic activity is improved. The electrical conductivity equation is as follows:

3 *κ* = 1/*ρ* = 1/(*C*Δ*Φ*/*I*)

here, *C* is the probe coefficient, which is dependent on the arrangement and space between the four probes. According to the four-point probe method, we measured the voltage under different currents and calculated the average value; the values of resistivity (*ρ*) and conductivity (*σ*) were calculated for the Pb/Sn/Al composite and Pb–Ag alloy, and the results are shown in Table 4.

**Table 4:** Conductivity (*σ*) of the composite materials under different processing conditions

No.	Pb/Sn/Al composite			Pb–Ag alloy		
Current value/A	0.1	0.3	0.5	0.1	0.3	0.5
*σ*/10^6^ Ω^−1^ m^−1^	0.095	0.125	0.140	0.038	0.043	0.049

After entering the tested voltage into eqn (3), the electrical conductivity of the Pb/Sn/Al composite (0.12 × 10^6^ Ω^−1^ cm^−1^) was found to be higher than that of the Pb–Ag alloy (0.044 × 10^6^ Ω^−1^ cm^−1^), which was due to the addition of Sn and Al sheets. It is known that the electrical conductivity of metal-based composites is realized by the transition of free electrons; however, the transition became weak due to the scattering at the interface, which led to low electrical conductivity. For the Pb/Sn/Al composite, its interface consisted of two parts: the area at the Pb side where α-Pb and β-Sn solid solutions coexisted and a PbSnAl metallurgy-bonded area at the Al side. From the mixed law of metal-based laminated composites, the empirical formula can be used to estimate the electrical conductivity of Pb/Sn/Al laminated composite.^32^

4 *κ* = ∑*κ*_*i*_*V*_*i*_

here, *κ*_*i*_ and *V*_*i*_ are the electrical conductivity and volume fraction of each component, respectively.

According to the previous observation, the PbSnAl metallurgy layer (with a thickness of about 3.4 μm) at the Al side was too thin, which can be ignored for the electrical conductivity of the Pb/Sn/Al laminated composite. However, the area at the Pb side where α-Pb and β-Sn solid solutions coexisted significantly contributed to electrical conductivity. Ocak^33^ reported the effects of different contents of Sn on the electrical conductivity of Pb–Sn alloys, suggesting that the electrical conductivity of Pb–Sn alloys (the highest value was about 0.092 × 10^6^ Ω^−1^ cm^−1^) is much higher than that of pure Pb (0.045 × 10^6^ Ω^−1^ cm^−1^). Briefly, the sandwich laminated structure of the Pb/Sn/Al laminated composite can effectively improve its electrical conductivity.

**Fig. 7:**
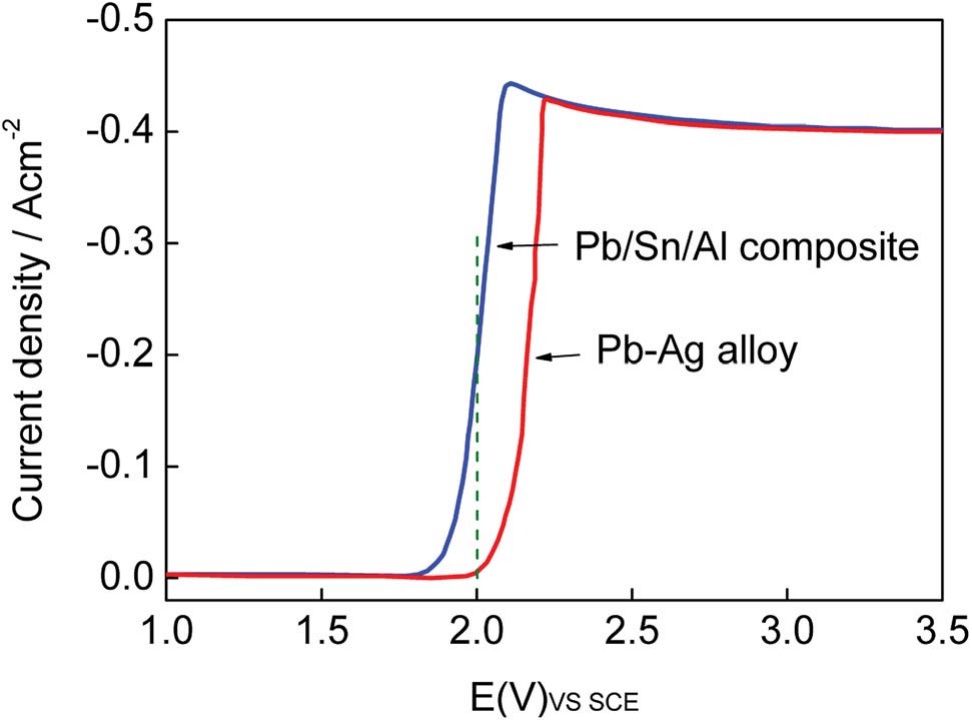
Linear scanning voltage–current curves of the Pb/Sn/Al composite and Pb–Ag alloy.

The LSV curve of the Pb/Sn/Al composite shifted to left in comparison with that of the Pb–Ag alloy electrode (as shown in Fig. 7). At the same electrode potential, the electric current density of the Pb/Sn/Al composite was higher than that of the Pb–Ag alloy electrode. According to electrochemical kinetics,^34^ a steady polarization curve can efficiently reflect the relation between the reaction velocity of the electrode and electrode potential. It can be seen that the reaction velocity of the Pb/Sn/Al composite electrode was higher than that of the Pb–Ag alloy electrode, which indicated that the electrode reaction process of the Pb/Sn/Al composite easily occurred at the same electric current density. In this case, positive charge was less accumulated at the reaction interface with a lower polarization potential of the Pb/Sn/Al composite, which reduced the energy of the electrode reaction and improved the electrocatalytic activity of the electrode. The experimental results were in good agreement with the previously reported results,^35^ where the improvement in the conductivity of an electrode material can affect the electrocatalytic activity of the electrode. A lower polarization potential indicates faster reaction velocity; thus, the electrocatalysis of the Pb/Sn/Al composite was high enough to reduce the bath voltage in the process of zinc deposition, which was probably due to the high electrical conductivity of the Pb/Sn/Al composite, according to the above-mentioned analysis.

## 4. Conclusion

In summary, a Pb/Sn/Al laminated composite anode material was prepared *via* vacuum hot-pressing welding. The addition of Sn was believed to remarkably solve the immiscibility between Pb and Al sheets, resulting in metallurgical bonding at the interface, where large block α-Pb and a small amount of β-Sn solid solutions were formed. Thus, the superior mechanical strength of the Pb/Sn/Al laminated composite electrode material in comparison with that of the Pb–Ag alloy was due to the sandwich structure of Pb/Sn/Al as well as its compact interface. Correspondingly, the sandwich laminated structure can effectively improve the electrical conductivity of the Pb/Sn/Al laminated composite. The electrocatalysis of the Pb/Sn/Al composite was high enough to reduce the bath voltage in the process of zinc deposition. Therefore, the preparation of Pb/Sn/Al laminated composite anodes has provided a good approach to improve the mechanical properties of Pb alloy anodes.

## Conflicts of interest

There are no conflicts to declare.

## Supplementary Material
